# Unlocking the power of LiOH: Key to next-generation ultra-compact thermal energy storage systems

**DOI:** 10.1016/j.heliyon.2024.e33992

**Published:** 2024-07-04

**Authors:** F. Achchaq, S.-C. Moon, P. Legros

**Affiliations:** a University of Bordeaux, CNRS, Bordeaux INP, I2M, UMR 5295, F-33400 Talence, France; b Arts et Metiers Institute of Technology, CNRS, Bordeaux INP, Hesam University, I2M, UMR 5295, F-33400 Talence, France; c School of Mechanical, Materials, Mechatronic and Biomedical Engineering, University of Wollongong, NSW 2522, Australia; d University of Bordeaux, CNRS, PLACAMAT, UAR 3626, F-33600 Pessac, France

**Keywords:** Alkali metal hydroxide, PCM, Enthalpy-temperature function, Softening, Anisotropy, Thermal property, Ultra-compact TES

## Abstract

This study explores the potential of untapped lithium hydroxide (LiOH) as a phase change material for thermal energy storage. By overcoming the challenges associated with the liquid LiOH leakage, we successfully thermal-cycled LiOH in a laboratory scale experimentation, and observed its stability (>500 thermal cycles), without chemical decomposition. This step has never been performed to date. Its solid-to-liquid reversible transitions temperatures and related solidification/melting enthalpies values have been verified. Then, the first experimental characterization of LiOH's thermal properties shows unexpected values for its heat capacity, thermal conductivity and diffusivity, in contradiction with the few ones available in literature. This opens avenues for LiOH's applications for the storage of sensible and latent heat, as shown through the increased cycle efficiency potential of a thermal energy storage system if based on its energy storage capacity; up to six times more volumetric energy density compared to traditional Solar Salt-based systems used in the solar tower plant (4.5 GJ/m^3^ vs. 0.76 GJ/m^3^ over 1000 thermal cycles). Additionally, we observed a softening phenomenon that occurs inconsistently during heating, but which may account for its excellent melting properties and the interplay with other raw chemicals. This new insight contributes certainly to the underlying mechanisms in the synthesis of another promising heat storage material in development: the peritectic compound Li_4_Br(OH)_3_. This pioneering work suggests LiOH as a promising ultra-compact thermal energy storage material for filling the intermediary gap from current to next-generation solar power plants, although its large-scale application requires further investigation to achieve economic viability.

## 1 Introduction

### 1.1 Importance of developing new phase change material (PCM) in industrial perspective

Concentrating Solar Plant (CSP) technologies such as parabolic-trough, power tower, linear Fresnel, and dish Stirling CSPs harness direct solar radiation for thermal energy production and electricity generation. By integrating CSPs, particularly parabolic-trough CSPs and solar power towers, with Thermal Energy Storage (TES) systems, the annual capacity factor of solar plants has substantially improved. The energy output increase from 30 % up to 50 %, compared to 20–25 % without TES was reported [[1], [2], [3], [4]]. Despite these advantages, broad application of CSP technologies remains as a challenge due to certain limitations of the heat storage materials currently in use [5]. For instance, molten salts are widely used as a Heat Transfer Fluid (HTF) regardless their low efficiency in heat transfer. Although liquid sodium has recently gained attention as a potential HTF thanks to its high thermal conductivity (140 W/m.K), low vapour pressure, and thermal stability up to 575°C [6,7], the solar power industry has been exploiting only the sensible heat using these materials, overlooking the potential advantages of phase change materials (PCMs).

PCM-based TES systems were proposed as early as the 1970s–1980s, but their application in CSPs is still very limited for high temperature applications. The challenges of PCMs with multi-components salt-based heat storage materials such as Solar Salt, HITEC, and HITEC XL are low thermal conductivity, inferior to 1 W/m.K, non-congruent behaviour leading to material degradation, and difficulties in maintaining their energy stability over long-term thermal cycling. These issues limit the final performance of PCMs in TES systems [8,9]. There have been numerous proposals based on both experimental and simulation studies for improving the thermal conductivity of these materials. It includes the development of multi-component alloys with added particles of various natures or encapsulated PCMs, from nano-to micro- and macroscales, and numerical studies based on molecular dynamic methods for salt-based nanofluids development for instance [[8], [9], [10], [11], [12], [13]]. In addition, the long-term thermal cycling issues still remain. Indeed, the number of consecutive thermal cycles applied to phase change materials for heat storage applications at high temperatures, without added particles or matrix host, are very limited. For instance, the number mentioned in Ref. [14] varies between 3 and 5 only, but considered as sufficient for exhibiting an “excellent thermal cycle stability”. However, Wang et al. showed for the same ternary eutectic compound (LiNO_3_–NaNO_3_–KNO_3_) that its thermal stability is “excellent *at short-term”* only, meaning for 5 consecutive thermal cycles (heating/cooling). The stability is no more guaranteed at long-term due to the leakage issue during experiments. “Long-term experiment” means here keeping the salt at liquid phase isothermally for 20 h [15]. The solutions based on matrices impregnated with molten salts and nano-enhancement technology are promising but they also often compromise the amount of available thermal energy, significantly increasing the final cost of the storage system and maintenance requirements [12,14].

This study aims to bridge the gap between the combined heats exploitation (sensible and latent). We propose the investigation of unary salt-based PCMs that can overcome the segregation issues often occurring in multi-component materials. Following comprehensive literature survey, investigation of thermophysical properties, and thermodynamic modelling, we recognized pure Lithium Hydroxide (LiOH) as a highly promising candidate for PCM thanks to its desirable properties in thermal conductivity and thermal stability. In this study, we focus on understanding the thermal behaviour of LiOH during solid ⇄ liquid transitions, assessing performance of LiOH as a PCM. The possible issues for industry scale application such as volume expansion/shrinkage during phase change, leakage and degradation under long term application are also addressed.

### 1.2 Literature survey and theoretical study to find the best PCMs

**Fig. 1:**
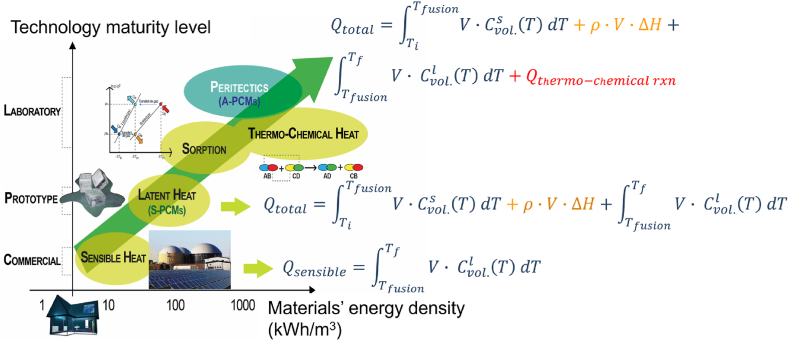
Technology maturity of the heat storage materials according to their potential energy density and to the kind of material heat exploitation (sensible heat terms are in blue, latent heat ones in yellow and the thermochemical in red). (For interpretation of the references to colour in this figure legend, the reader is referred to the Web version of this article.)

Persistent efforts are undertaken to identify optimal standard PCMs (S-PCMs), *-i.e*. materials that possess the highest possible energy density through liquid/solid transition and the ability of stable charging/discharging over the system's operating temperature range [13]. To locate PCMs that meet these criteria and exhibit pure compound-like thermal behaviour as eutectic compounds (substance mixtures with lowest possible melting points), screening was performed among all binary compounds exhibiting invariant reaction at fixed temperature. This includes monotectic-, eutectoid-, peritectic-based PCMs, among others. By analysing phase diagrams, calculated using the thermodynamic modelling software FactSage 7.0® previously detailed and parameterised in Ref. [14], we concluded that stoichiometric peritectic compounds generally exhibit a latent heat two to three times greater than that of S-PCMs. The performance of these advanced PCMs (A-PCMs) increases by 30–45 %, depending on the considered compound. This increase is directly associated with a reversible chemical reaction that occurs at a constant temperature, coupled with latent heat during the solid ⇄ liquid transition (Fig. 1) [14]. Through theoretical analysis, the salt-based binary peritectic compound Li_4_Br(OH)_3_ was recognized as a promising candidate, experimental work was then carried out to develop the synthesis protocol of Li_4_Br(OH)_3_ [15]. The first results as well the discovered enthalpy and polymorph structures of Li_4_Br(OH)_3_ are encouraging, leaving the need for further investigation [16].

**Fig. 2:**
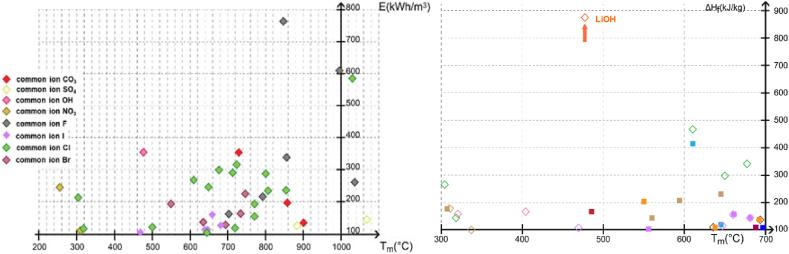
Theoretical assessment of the unary compounds according to a/the volumetric energy density in the 200–1100 C range, and to b/the related enthalpy in the 300–700 C range gathered from literature (full squares) and from FactSage 7.0® database (empty squares).

The need of novel TES systems that can be fastly implemented for a market deployment has been a strong incentive to reconsider unary compounds from the same chemical family. Indeed, the phase separation issues, frequently seen in alloy systems can negatively impact thermal conductivity and heat transfer when operating conditions are not precisely maintained. Among the various salts studied, LiOH exhibits a high latent enthalpy value of 875 kJ/kg at 477°C. It appears thus that this material suits the ideal condition for steam turbine system, and is a promising candidate for efficient thermal energy storage (Fig. 2).

The thermal properties of LiOH were collected from the available literature, particularly those using differential scanning calorimetry (DSC) (Supplementary Tables 2 and 3) [8,17]. The reported melting point of LiOH varies significantly, ranging from 413°C to 477°C, and the reported melting enthalpy, ranges between 466 and 1111 kJ/kg. For example, Fernandez et al.‘s recent work [18] on LiOH has focused on melting and solidification temperatures, with a reported melting temperature of 413.1°C. This value contradicts with not only a melting temperature of 462°C in provider's specification but also other values found in various literatures. Furthermore, information regarding the solidification point is notably absent. Confusions in interpreting the observations such as a ‘softening’ or solid ⇄ solid transition add to the ambiguity [19,20]. LiOH's thermal property data in both solid and liquid phases, such as thermal conductivity and diffusivity are barely available. The discrepancies and the lack of information about LiOH properties necessitate the demand and direction of research recommended in Ref. [8] and underscores more comprehensive studies for LiOH.

The most extensive thermal characterization of LiOH dates well back to 1976 [21], but there is no available data on the energy stability of LiOH under thermal cycling condition. This is mostly due to the difficulties in retaining sample within a crucible over the extended experiment period. Issues encountering during experimentation and possible in-plant application of LiOH include its decomposition into Li_2_O, reactivity with CO_2_, corrosive nature, and the ‘creeping’ phenomenon observed in its liquid phase. To circumvent this, we sealed the crucible using a house-built welding process allowing us to study LiOH's energy stability.

## 2 Material and methods

The Differential Scanning Calorimeter (DSC Sensys 3D Evo, provider: SETARAM) was calibrated using the stainless steel crucibles and indium, tin, lead, zinc, and aluminum as standard materials with purities of 99.99 % or greater. These standard materials cover the phase transition points from 156.6°C (related enthalpy: 28.57 kJ/kg) to 660.3°C (related enthalpy: 400.1 kJ/kg). The device was then calibrated at a heating and cooling rate of 0.5°C/min and of 10°C/min over a temperature range corresponding to the protocols applied then for the enthalpy-temperature function determination and for the thermal cycling study.

^1^*Enthalpy-temperature function determination*

Anhydrous LiOH powder sample (98 % purity) was supplied by Acros Organics. About 60 mgLiOH samples were prepared in a glove-box with an argon-controlled atmosphere to prevent oxidation and hydration. Sample was weighed using a Mettler XP6U ultra-microbalance (precision = 0.02 mg), and then placed in a sealed Inconel 618 crucible. Following the calibration of DSC, the sample was placed inside the DSC furnace. Inert atmosphere by flowing 30 mL/min of dry argon was maintained within the crucible and experiments were performed in triplicate in isothermal step scan mode. To establish the LiOH enthalpy-temperature function within the range of 230–515°C, a heating scanning rate of 0.5°C/min for each temperature increase of 1 C was applied. At each increment, a holding time of 60 min was applied to ensure thermal equilibrium before reaching the maximum value of 520°C. A cooling-to-room temperature at a scanning rate of 10°C/min was then applied.

^2^*Thermal cycling*

For thermal cycling experiments, two 90 mg LiOH samples were prepared from two different batches, and each sample was placed in a stainless steel crucible. The temperature cycling between 250 and 545°C was chosen to evaluate the thermal and enthalpy stability of LiOH over longer exposure to operational condition. The same heating and cooling scanning rates of 10 K/min was applied holding for 15 min at each minimum and maximum temperature. For the first sample, 170 times of thermal cycling were performed. During this thermal cycling, the maximum temperature value was incremented from 530 to 535, and to 545°C to investigate the enthalpy stability over cycle numbers. Then, the maximum values were increased up to 640°C over 67 added cycles to find out the LiOH degradation temperature that corresponds to 640°C. To maintain a significant safety margin, the maximum temperature value of 545°C was applied to the second sample over 540 thermal cycles. A same heating and cooling scanning rates of 15°C/min were firstly applied to the 235 cycles, and then the same value of 10°C/min was applied to the left 305 thermal cycles.

^3^*Chemical decomposition checking*

Powder X-ray diffraction (XRD) equipment, PANALYTICAL X'PERT 3 Powder diffractometer using MoK α radiation (λ = 0.71073 Å) was used to analyse crystal structure of LiOH samples used in the preceding DSC experiments. Owing to the hygroscopicity of the salt samples, they were ground and mounted in capillaries within an argon-filled glove box to prevent atmospheric contamination.

^4^*Thermal properties determination*

The hot disk reference method detailed in Ref. [22] has been used to assess LiOH's thermal properties. Cuboid-shaped LiOH samples were prepared with 15 mm in thickness and 40 mm for sides. Samples were polished using 4000 grit paper to maximize contact between the material and thermal sensor. Thermal conductivities were then measured both in-plane and normal to the plane using the hot disk technique. The thermal sensor, which functioned as both heat source and temperature sensor, was placed between two pieces of the sample and heated by electrical current.

^5^*Morphological observations*

Using optical and IR cameras during thermal cycling, sample morphology change was monitored *in situ*. Microscopic morphology was also observed using a Scanning Electron Microscope (SEM; Zeiss 50 Evo) under secondary vacuum (10^−4^ Pa) in secondary electron mode.

## 3 Results

### 3.1 LiOH transition temperatures, enthalpy and specific heat capacity

**Fig. 3:**
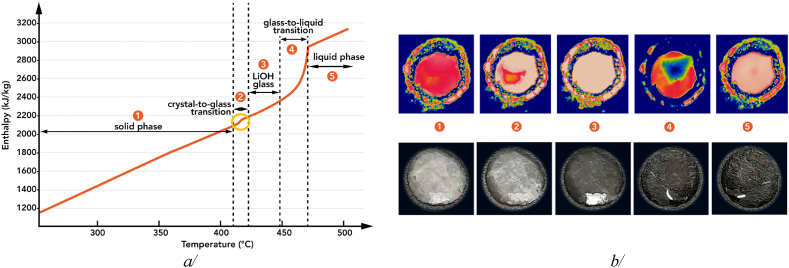
a/Experimental LiOH enthalpy - temperature function obtained at ambient pressure under equilibrium conditions; the yellow circle highlights the solid-solid transition, and b/IR snapshots at the top and optical ones at the bottom explaining the non-conventional obtained enthalpy - temperature function for this pure compound. (For interpretation of the references to colour in this figure legend, the reader is referred to the Web version of this article.)

DSC step scan mode experiments revealed a significant solid ⇄ solid transition at 413 C and a melting point at 471 C, accompanied by an enthalpy change of 853 kJ/kg as shown in Fig. 3a. These results are reasonably consistent with earlier research [21], but the enthalpy-temperature function shape of LiOH in our measurement diverges from theoretical predictions, resembling that of the stoichiometric peritectic compound Li_4_Br(OH)_3_ [23]. These findings hint at LiOH's thermal behaviour being more complex than previously anticipated. Interestingly, a transition of LiOH morphology from white crystalline to transparent substance prior to melting has been observed using both optical and IR cameras during thermal cycling (Fig. 3b). Pertinent to our experimental condition and resulting observations, it is highly unlike that the formation of a mono-crystalline structure could occur, and probably the latter structure could be an amorphous phase. Being different from standard behaviour exhibited by pure compounds, this solid ⇄ solid transition or ‘softening’ behaviour during heating seems to be a unique characteristic of LiOH. This ‘softening’ could make LiOH's melting easier by lowering the energy barrier associated with its crystal structure, confirming the observation and hypothesis of other earlier studies [19,20,23] that used the term ‘softening'.

**Fig. 4:**
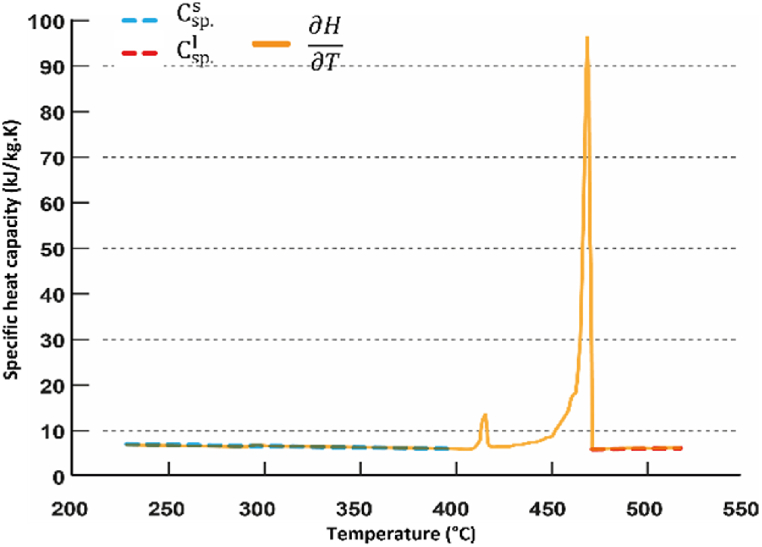
Evolution of the LiOH specific heat with temperature, obtained at ambient pressure under equilibrium conditions (yellow line) with the specific heat at solid phase calculated between 230 and 390 C (dashed blue line) and the specific heat at liquid phase just after complete melting (471°C) up to 520°C (dashed red line). (For interpretation of the references to colour in this figure legend, the reader is referred to the Web version of this article.)

Lastly, the specific heat capacity $C_{p}$ as a function of temperature is shown in Fig. 4. As it can be seen, the solid-solid transition event at 413°C is obvious. The average specific heat capacity of solid-phase LiOH is 5.91 kJ/kg.°C obtained by a linear equation,$C_{p}^{s}$ = −0.005613 *T* + 7.855 in the 230 ≤ T ≤ 390°C range. The average specific heat capacity of LiOH liquid phase is 5.65 kJ/kg°C in the temperature range from 471 to 520 °C.

The high specific heat capacity of solid-phase underscores LiOH's potential as a material for sensible heat storage, particularly when compared to the specific heat capacity range of refractories (0.70–1.2 kJ/kg°C) over a 0–1500°C temperature range. 1 kg of solid-LiOH in a temperature range of 21–450 C, will store/release ∼2.5 MJ of sensible heat, which is five times greater than the energy that 1 kg of fireclay-based refractory material can carries over the same temperature range. Intriguingly, our confirmed specific heat capacity for solid-phase LiOH is nearly twice as high as studied by Janz et al. in Ref. [24] and in Gurvich et al. in Ref. [25] using DSC standard mode. This discrepancy is also observed when compared with values found in earlier report by Tye et al. [21], where the standard drop calorimetry method was employed (Table 1). The lower measurement of specific heat capacity from earlier studies compared to our measurement might be attributed to imperfect sealing to completely prevent leakage during the experiment, leading to a significant enthalpy decrease also. In our experimentation, the leakage of LiOH associated with creeping phenomena well above melting point has been prevented by implementing special sealing (see Section Material and methods-*Thermal cycling*).

**Table 1:** Anhydrous LiOH thermal properties from Tye et al. [21], the FactSage software [26][ and this work.

	Tye [20]				
	Reagent 99.8 %	Commercial 95.0 %	This work Reagent 98 %		FactSage 7.0® n.a
$T_{m}$(°C)	470	471	471		477
${\Delta H}_{m}$(kJ/kg)	1015	1110	1103		873
$\rho_{s}$(kg/m^3^)	1434 at 20°C	1376 at 20°C	1455 at 20°C		1460 at 20°C
	1392 at 450°C	1330 at 450°C	–		-
$\rho_{l}$(kg/m^3^)	1346 at 560°C	1360 at 560°C	1426 at 545°C		1342 at 550°C
$C_{sp.}^{s}$(kJ/kg.K)	3.15 at 400°C	3.05 at 400°C	5.91		n.a.
$C_{sp.}^{l}$(kJ/kg.K)	3.94 at 550°C	4.15 at 550°C	5.65		n.a.
$k_{s}$(W/m.K)	2.50 at 50°C	3.06 at 50°C	$k_{∥}$ ∼ 44	$k_{⊥}$∼0.86 at 20°C	n.a.
	1.65 at 200°C	2.00 at 200°C	–		n.a.
	1.27 at 400°C	1.37 at 400°C	–		n.a.
$\alpha_{s}$(x 10^−6^ m^2^/s)	n.a	n.a	$\alpha_{∥}$ ∼ 5	$\alpha_{⊥}$∼0.10 at 20°C	n.a.
$k_{l}$(W/m.K)	0.85 at 490°C	0.85 at 490°C	–		n.a.
	0.88 at 600°C	0.89 at 600°C			n.a
$\alpha_{l}$(m^2^/s)	n.a	n.a	–		n.a

### 3.2 LiOH energy stability

**Fig. 5:**
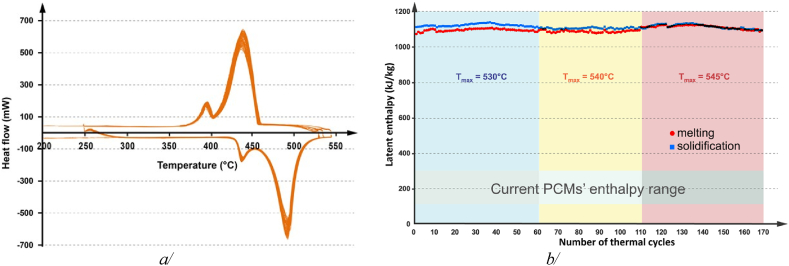
a/LiOH thermograms obtained, in DSC standard mode, for 170 thermal cycles, at 10 K/min for both heating and cooling steps, b/Related melting and solidification enthalpy values with the different applied maximum temperature values.

Fig. 5a/ presents the behaviour of the heat flux over 170 thermal cycles between 250 and 545°C for a LiOH sample, with a consistent scanning rate of 10 K/min for both heating and cooling. The ‘softening’ phenomenon detected in DSC step scan mode experiments along with visual observations using optical and IR camera, also consistently appears during thermal cycling, confirming the reversibility of softening and the stability of the material's heat storage properties. This finding greatly enhances the understanding of the durability and longevity of LiOH-based thermal energy storage systems. It's interesting to note that with the significantly higher scanning rate than used in enthalpy determination experiment, the occurrence of softening during heating underwent ∼15°C superheat. The melting and solidification enthalpy values, which can be obtained from the peak area, are represented as a function of the thermal cycles number as shown in Fig. 5b. The melting and solidification enthalpies remain remarkably identical, especially after adjusting the maximum temperature value to 545°C. When the maximum temperature is too low, the heat distribution within the system is indeed not finished, leading to slight decrease in calculated peak area for melting enthalpy. Nonetheless, an average enthalpy of 1103 ± 14 kJ/kg and 1115 ± 15 kJ/kg are observed for melting and solidification respectively, completely in line with the values reported by Gurvich et al. in Ref. [25].

**Fig. 6:**
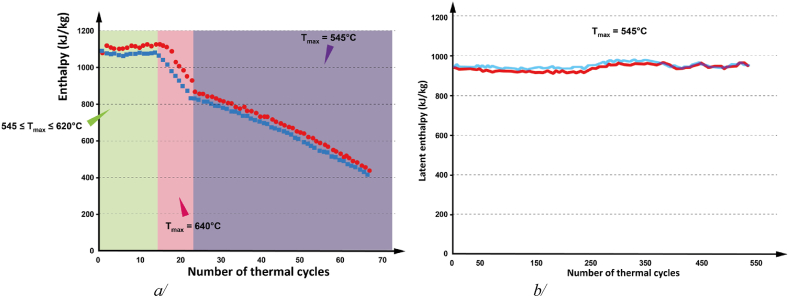
a/First batch LiOH melting and solidification enthalpy values for 67 thermal cycles, at 10 K/min for both heating and cooling steps, b/second batch LiOH melting and solidification enthalpy values for 235 thermal cycles, at 15 K/min for both heating and cooling steps, and then for 305 thermal cycles at 10 K/min for both heating and cooling steps.

Fig. 6a/ shows the obtained results when the maximum temperature is increased up to 640°C. The rapid decrease of enthalpy at 640 C observed is directly linked to a LiOH's decomposition phenomenon. Once initiated, the decomposition is irreversible. Other experiments were then carried out with LiOH samples from another batch of the same provider (see Section Material and methods-*Thermal cycling*). The melting and solidification enthalpy values are also represented as a function of the thermal cycle number shown in Fig. 6b. The applied scanning rate of 15°C/min to the 235 first thermal cycles implies the same slight discrepancy between the melting and solidification enthalpy values, for the same reason. As previously, the melting and solidification enthalpies remain remarkably identical, especially when the scanning rate temperature is fixed to 10 C/min. An average enthalpy of 960 ± 13 kJ/kg and 951 ± 11 kJ/kg are observed for melting and solidification respectively, which represents a variation around 15 % with the previous experiments. For these second batch samples, the consistent ‘softening’ phenomenon detected previously in DSC step scan mode experiments does not appear at all during thermal cycling. This time, the obtained thermograms are as expected from a pure compound, *-i.e.* with one melting peak and one solidification peak. Further investigation is needed to establish a link, if any, between the absence of the ‘softening’ peak and the slight decrease in enthalpy values observed, and to understand why this thermal behaviour difference from two LiOH batches of the same provider.

### 3.3 LiOH density, volume expansion, thermal conductivity and diffusivity

**Fig. 7:**
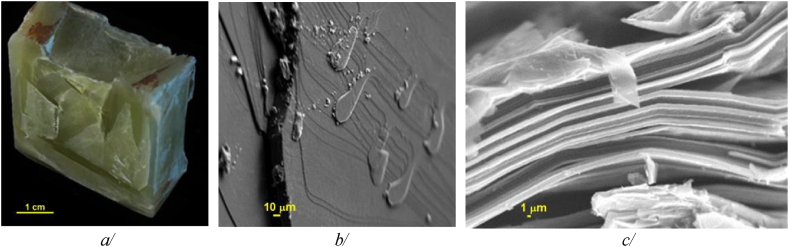
LiOH morphology at a/macroscale (by optical camera), and at microscale (by SEM) showing the lamellar structure b/in a planar direction, and c/in normal to the plane.

There has been a good agreement in density of LiOH at room temperature between measured values [21,27] and calculated value using FactSage 7.0® [26]. Interestingly, our estimated density decreases by only 2 % during transition from solid to liquid phase, while volumetric expansion during heating in other salts is ∼10 %. This LiOH's specific behaviour is highlighted as one of contributing factors for LiOH being powerful compound. Microscopic observations of LiOH revealed very thin, evenly distributed, and tightly packed lamellae as shown in Fig. 7. This provides insights into its thermal conductivity and diffusivity. This suggests anisotropic heat transfer likely taking place along two preferential directions: in-plane (plane of lamella) and normal to the plane of lamella. The hot disk reference method detailed in Ref. [22] has been used to assess LiOH's thermal properties. The observed morphology exhibits an apparent anisotropy. In solid state, LiOH's thermal conductivity was measured to be 43.97 ± 2.973 W/m.K and 0.856 ± 0.0400 W/m.K in the radial (in-plane) direction and normal to the plane respectively, and the diffusivity was measured to be 5.18 ± 0.350 × 10^−6^ m^2^/s and 0.101 ± 0.00399 × 10^−6^ m^2^/s in the radial (in-plane) direction and normal to the plane respectively. This strong anisotropy mostly attributed to the lamellar structure, in which there is a significantly increased heat flow resistance normal to lamellar plane direction.

### 3.4 XRD analysis

**Fig. 8:**
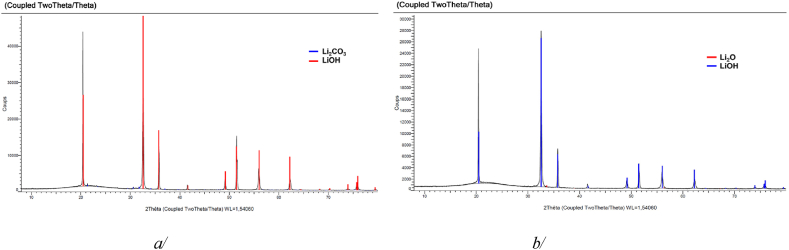
a/XRD patterns of LiOH obtained a/before the thermal cycling experiment and b/at its end.

Fig. 8 shows XRD patterns obtained for the sample before and after the 545 thermal cycles. No decomposition occurred for the applied operating conditions used for this experiment. More generally, no decomposition occurred as long as the maximum temperature did not achieve 640°C or no leakage took place.

It is worth mentioning that, as expected, XRD patterns do not allow distinguishing between the LiOH samples from the different batches: the ‘softening’ phenomenon cannot be visualised with this method.

## 4 Discussion

### 4.1 Softening

**Fig. 9:**
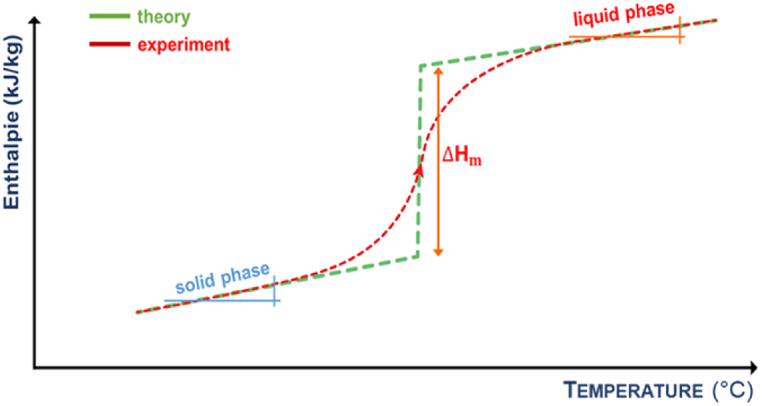
Standard enthalpy-temperature function of pure compounds.

First batch LiOH's enthalpy-temperature function (Fig. 3a) varies from that of general unary substances such as water, silver, or copper, which undergo a single solid-to-liquid transition (Fig. 9) [28]. This deviation could account for the inconsistencies in melting temperature indicated in Fernandez et al.‘s study [18].

The understanding of the ‘softening’ phase process presents promising prospects for thermal energy storage materials knowledge. We recognize the need for further research to fully understand this phenomenon. From an energy efficiency perspective, the softening could reduce the energy needed to overcome the crystal lattice's rigidity prior to melting of LiOH. This could lead to a more energy efficient storage and retrieval process. Moreover, the similarity of the enthalpy-temperature function between LiOH and Li_4_Br(OH)_3_ peritectic compound suggests LiOH could play a vital role in the structural formation of Li_4_Br(OH)_3_ the enthalpy-temperature function of Li_4_Br(OH)_3_ can be found in Ref. [15]. Our previous study indicates that in the absence of this softening phase the formation of less energy polymorphic structure is favoured [16]. This highlights the importance of LiOH's softening transition for energy storage capabilities in LiOH family materials. The softening transition of LiOH might be vital for stabilizing this Li_4_Br(OH)_3_ peritectic compound under thermal cycling conditions. For moving towards development, understanding the intricate thermal behaviour of LiOH and Li_4_Br(OH)_3_ is therefore of paramount importance.

### 4.2 Anisotropic property

Thermal conductivity of LiOH normal to plane direction is comparable to that of typical insulator such as glass or concrete, while its in-plane thermal conductivity exceeds even that of superior thermal conductive materials such as Inconel or lead (18 and 35 W/m.K, respectively). A question is risen that how the orientation of plane (lamellae) is determined during the samples manufacturing. There will be a great potential to exploit this anisotropy in designing and optimizing step in the process of developing TES systems. This could potentially lead to optimum design that is much more efficient and adaptable for varying circumstance in thermal storage and/or regulation applications. For instance, in the case of solar field, exploiting the highest thermal conductivity of the material by developing an appropriate storage container would avoid the use of a heat exchanger with complex geometry. Obviously these will require sophisticated numerical modelling to predict their performance under various operating conditions.

Table 1 gathers the data from the most complete study on LiOH found in literature as far as we know, with the data of this work and the ones extracted from the thermodynamic modelling software database.

Tye et al. [21] mentioned neither the ‘softening’ phenomenon nor the anisotropy of the thermal properties in their work, as well the thermodynamic modelling database.

### 4.3 Storage container

Given that LiOH is a strong base, when enough time at elevated temperatures is given, it can react with materials in contact. This could be a concern depending on container material, as LiOH may degrade the tank's structural integrity by reacting to form a lithium metal oxide. To overcome this issue, a storage tank with a protective coating can be utilised. Another way may consist in allowing a passivation layer to form over time. Research to assess this last assumption is going on. Another factor to consider is the thermal stress experienced by the storage tank during operational thermal cycling. Over time, repeated thermal stress (fatigue) can cause its structural failure. This necessitates the proper design of container tank in industry scale. Pertinent to our laboratory experiment up to 545 cycles (equivalent to 1 year and half full time operation), there was no sign of failure, such as a sudden drop in the amount of LiOH, unexpected change in the heating curves, or visible sign of deterioration in the crucible. The results open questions for future research as what strategy for storage container is required together with LiOH management. Current results confirm that it withstands the temperatures commonly encountered in thermal storage applications without degrading or loosing energy storage capacity. This satisfies also the crucial criteria as a storage material under appropriate operational conditions, while giving the potential for greatly increased cycle efficiency in a thermal energy storage system. This study is currently used for the design and the conception of a prototype LiOH- based thermal battery in order to bring the proof that it can work for a market deployment.

### 4.4 Proposal of a LiOH-based ultra-compact energy storage system

LiOH's superior thermal stability, particularly its unique ‘softening’ behaviour at 413°C, differentiates it from other conventional thermal storage materials, highlighting its potential as a novel material for long-term applications in energy storage systems. This could be especially valuable in the context of intermittent renewable energy sources, such as solar and wind power, where energy must be stored during periods of high production for use during times of lower production. The latent heat alone is sufficient to provide the totality of the sensible energy stored/released by Solar Salt in liquid sensible mode. This could significantly increase the efficiency and cost-effectiveness of thermal energy storage systems. Solar Salt is taken here as a reference, for this is the most studied heat storage material, belonging to the salt family. Solar Salt is still used at market level in the TES system of current solar plants. Based on our results, it is obvious that LiOH possesses superior heat storage capabilities compared to traditional storage systems like Solar Salt. Between 20 and 545°C, LiOH can store up to 6.1 GJ/m³ of heat (∼1.7 MWh/m^3^), in sensible and latent forms. In case the lower limit of cycling temperature is set to 200°C, the capacity is reduced to 4.5 GJ/m³, still outperforming Solar Salt system, which only stores 0.76 GJ/m³ of heat in the sensible form in current operational temperature range, 290 and 555°C. The benefit of increasing thermal stability up to 600°C of current Solar Salt by using a complex technology that has been recently considered by Bonk et al. [29] is minor (only reaches 0.91 GJ/m^3^) compared with LiOH capacity. Even with considering the additional latent heat storage functionality in Solar Salt proposed by Costa et al. [30] for an Organic Rankine Cycle (ORC) turbine, the benefit is only the addition of 0.2 GJ/m³ for the latent heat portion at ∼220°C in addition to 1.1 GJ/m³ for sensible heat between 220 and 555°C. Besides, implementing this method encounters challenges, it does not solve issues such as segregation problems and a significant volume expansion of ∼10 % during the transition between solid and liquid states.

**Fig. 10:**
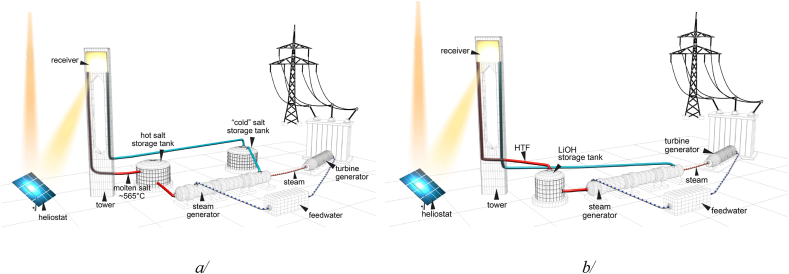
a/Schematic configuration of the current commercial solar tower power plants, b/proposed solar power plants utilizing heat storage system based on LiOH. This requires a single ultra-compact tank that replaces complicated two tank system in the current solar tower power plants.

Using LiOH offers a promising solution for designing a highly robust, ultra-compact, and ultra-efficient thermal energy storage system. One potential design for this could be a single-tank system, using LiOH as the storage medium and liquid sodium as the heat transfer fluid (HTF). This setup could overcome the limitations of current Solar Salt-like systems and offer a more efficient solution for storing and releasing heat. This new approach has a potential to revolutionise thermal energy storage, and could be a game changer for heat storage and conversion industries. Thorough scientific studies more in depth paralleling engineering consideration in industry perspective are needed to confirm the feasibility and optimise such a system in real-world application. Fig. 10 provides a visual representation of how such a system could be implemented.

## 5 Conclusions & perspectives

Thermal energy storage (TES) systems can significantly help the integration of renewable energy sources into the power grid, fostering economic benefits by reducing reliance on fossil fuels and stabilizing energy prices. This study have demonstrated how the thermodynamic properties, thermal stability, and anisotropic thermal conductivity of the salt-based phase change material (PCM) lithium hydroxide (LiOH) have the potential to revolutionise thermal energy storage. Indeed, LiOH melts invariably at 471°C and exhibits a very high latent heat varying from ∼950 to ∼1100 kJ/kg, depending on the occurrence, or not, of a softening phenomenon at 413°C. From the experimental enthalpy-temperature function, the unexpected average specific heat capacity of solid-phase LiOH obtained is of 5.91 kJ/kg°C and of 5.65 kJ/kg°C in liquid phase. Eventually, The material shows a strong thermal anisotropy. Its thermal conductivity value normal to plane direction is comparable to that of typical insulator such as glass or concrete (∼0.86 W/m.K), while its in-plane thermal conductivity (∼44 W/m.K) exceeds even that of superior thermal conductive materials such as Inconel or lead (18 and 35 W/m.K, respectively). Further investigations are currently on-going to understand the underlying mechanisms leading to these unusual features. These findings suggest the possibility of creating ultra-compact and highly efficient heat storage systems. Moreover, this LiOH-based TES system aligns perfectly with the Direct Steam Generation (DSG) process, a method that has been extensively researched since 1998 in the Plataforma Solar de Almeria (PSA). The implementation of this process into commercial plants has been awaiting the development of a suitable latent heat-based thermal storage system. The compact nature of the proposed LiOH-based system also allows for its potential application in heat recovery in various industries, such as metallurgy and nuclear energy industries. A better understanding of alkali-metal hydroxides-based PCMs, and their potential applications, can guide their smarter integration into a variety of systems. A thorough review for economic viability, policy implications, and environmental impacts is essential to fully evaluate the feasibility and overall impact of this technology. The promising findings of this study will encourage further research and could potentially lead to the large-scale implementation of LiOH-based thermal storage systems. This could profoundly impact the energy landscape, moving us closer to a future where renewable energy can effectively replace fossil fuels.

## Data avaibility

The authors are unable or have chosen not to specify which data has been used.

## CRediT authorship contribution statement

**F. Achchaq:** Writing – original draft, Validation, Methodology, Investigation, Formal analysis, Conceptualization. **S.-C. Moon:** Writing – review & editing, Validation, Methodology. **P. Legros:** Validation, Methodology, Investigation, Conceptualization.

## Declaration of competing interest

The authors declare that they have no known competing financial interests or personal relationships that could have appeared to influence the work reported in this paper.
