# Supplementary material for: Unlocking the power of LiOH: Key to next-generation ultra-compact thermal energy storage systems
Source: Heliyon. 2024 Jul 4;10(13):e33992. doi: 10.1016/j.heliyon.2024.e33992 (PMC11292522; doi:10.1016/j.heliyon.2024.e33992)
Supplement: Multimedia component 1 [file mmc1.docx]

**Supplementary Tables**

F. Achchaq^1, 2*^, S.-C. Moon^3^, P. Legros^4^

^1^ University of Bordeaux, CNRS, Bordeaux INP, I2M, UMR 5295, F-33400, Talence, France

^2^ Arts et Metiers Institute of Technology, CNRS, Bordeaux INP, Hesam University, I2M, UMR 5295, F-33400 Talence, France

^3^ School of Mechanical, Materials, Mechatronic and Biomedical Engineering, University of Wollongong, NSW 2522, Australia

4 University of Bordeaux, CNRS, PLACAMAT, UAR 3626, F-33600 Pessac, France

*^*^Corresponding email:* [*fouzia.achchaq@u-bordeaux.fr*](mailto:fouzia.achchaq@u-bordeaux.fr)

**Current scientific knowledge about LiOH**

The discrepancy observed for the other transition temperature values is assigned specifically to the presence of Li_2_CO_3_. This latter would react with LiOH and a eutectic compound would be formed, acting as an impurity with a fusion temperature occurring just before the melting of LiOH. The LiOH allotropic tetragonal modification mentioned in Gurvich’s work occuring between 397 - 413 °C, at ambient pressure, is not taken into account. Yet, anhydrous LiOH’s structure evolves according to the temperature at ambient pressure but it seems very difficult to explain the reasons why and how, as later mentioned by [1]. For some strange reason, when the raw material of one experiment shows “only traces of carbonate” with a transition temperature between 451 and 459 °C, [2] assigned it to a “pre-melting” of LiOH without more explanation and claims that there is no known “polymorphism” of LiOH. Yet, the fusion temperature values of the LiOH / Li_2_CO_3_ binary eutectic compound are reported to be of 431 °C in FactSage7.0^®^ and of 418.2 °C in the experiments performed by [3]. When reported in the works, the assigned values to this transition temperature are also scattered and they differ from the calculated and experimentally assessed ones. The listed values of the LiOH’s melting temperature vary from 462 to 477 °C, *-i.e.* a difference of 15 °C, while a variation from 466 to 923 kJ/kg is reported for the enthalpy of fusion. According to the analysis of [2], the most reliable data on LiOH are the melting temperature of 471 °C and the related fusion enthalpy of 875 J/g.

**Table 2.** Thermophysical properties of anhydrous LiOH in literature.

| **Used device** | **Purity**  **(%)** | **Transition temperature**  **(°C)** | **Melting temperature**  **(°C)** | **Transition enthalpy**  **(kJ/kg)** | **Melting enthalpy**  **(kJ/kg)** | **Reference** |
| --- | --- | --- | --- | --- | --- | --- |
|  | - | - | 462 ± 5 | - | 873 ± 15 | [Janz-1981] |
| Drop calorimeter | 96.6 | - | 473 | - | 878 | [Gurvich-1996] |
| Drop calorimeter | - | 451 - 459 | 471 | - | 875 |  |
|  | - | **-** | 462 | **-** | **-** |  |
| Ni-Cr-constantan thermocouple  immersed in melt | - | - | 477 | **-** | **-** |  |
| Differential Thermal Analysis (DTA) | - | 413 | 462 | **-** | **-** |  |
| DTA | - | 397 - 413 | 470 | **-** | **-** |  |
| DTA | - | - | 474 ± 1 | **-** | 923 |  |
| DTA | - | - | 473 | **-** | 848 |  |
| Visual method of thermal analysis | - | 397 - 410 | 477 ± 1 | **-** | - |  |
| Differential scanning calorimeter | 98 | 426 | 475 | 6.4 | 466 | [Kiat-1998] |
|  |  |  |  |  |  | - |
|  |  |  | 462 |  |  | [Hermann] |

A review of the works performed on LiOH reporting its transition phenomena has been thus carried out in order to clarify this point with a focus for the assessments using the differential scanning calorimeter (DSC). DSC is indeed renowned as being the most accurate device for the transition phenomena’ identification and the materials’ related thermophysical properties estimation. The LiOH’s data obtained during the heating step are gathered in Table 3. LiOH being very hygroscopic, it hydrates spontaneously at room temperature and ambient pressure if no precaution is taken into account, as demonstrated by [4]. For the same sample and the same operating conditions, the values of dehydration during the heating step differ from 40 °C depending on if the used crucible is open or sealed. The author noticed the reversibility of i) the (de)hydration reaction LiOH_(s)_ + H_2_O_(g)_ ⇄ LiOH∙H_2_O_(s)_, ii) a first order structural phase transition occurrence, at 425 ± 1 °C with a latent heat of 6.4 kJ/kg and iii) the lowest melting latent heat recorded value of ~ 466 kJ/kg. According to [5], the residual water presence appears with a related DSC peak at 77 °C for a heating rate of 5 °C/min, and this makes the latent heat of NaOH and of binary eutectic mixture with KOH and LiOH strongly decrease. No information is given for LiOH alone. At the end, the discrepancies in the obtained melting temperature values remains even by using the same technique, varying between 462 and 477 °C and no information is given about the solidification one. The solid -solid transition, also called “softening” [6], is barely mentioned and the LiOH melting enthalpy value varies also from 466 to 1111 kJ/kg.

**Table 3.** Reported transition phenomena of anhydrous LiOH in literature.

| **Used LiOH**  **and**  **crucible** | **Heating**  **rate**  **(°C/min)** | **Temperature range**  **(°C)** | **Dehydration temperature**  **(°C)** | **Softening temperature**  **(°C)** | **T_m_**  **(°C)** | **Softening enthalpy**  **(kJ/kg)** | **∆H_m_**  **(kJ/kg)** | **Ref.** |
| --- | --- | --- | --- | --- | --- | --- | --- | --- |
|  | - |  | - | - | 462 ± 5 | - | 873± 15 | [Janz-1981] |
| - purified and dehydrated; gold capsule | n.a. | 145 - 606 | - | 451 - 459 | 471 | - | 875 | [Gurvich-1996] |
| - reagent LiOH 99.8% | - | 300 - 600 | - | - | - | - | 1111 |  |
| - commercial 95% | - | 300 - 600 | - | - | - | - | 1015 |  |
| - Inconel capsule | n.a. | 124 - 940 | - | - | 473 | - | 894 |  |
|  | - | - | - | - | 474 ± 1  477 ± 1 | - | 923 |  |
| - gold container | 10 | - | - | - | 475  470 - 475 | - | - |  |
| - Merck; dehydrated | - | - | - | - | 473 | - | 849 |  |
| - OSI 98% stored at T_room_ with no precaution; sealed inox canister | 1 | T_room_ - 527 | 112 | 426 | 475 | 6.4 | 466 | [Kiat-1998] |
| - OSI 98% stored at T_room_ with no precaution; open inox canister under Ar atmosphere | 1 | T_room_ - 1397 | 72 | 425 | 472 | n.a. | n.a. | [Kiat-1998] |
| - Synthesized by the Bridgman technique | n.a. | n.a. | - | - | 470 ± 2 | - | - | [Baikov-2019] |
| - reagent LiOH 99% | 10 | 270 - 500 | - | - | 413.1 | - | - | [Fernandez-2022] |

Regarding the LiOH’s thermal properties, there is a greater lack of data. The very recent thermal properties presented in [7] deal with the melting and solidification temperatures only. The melting temperature of 413.1 °C is in complete contradiction with their provider that mentions a melting temperature of 462 °C, but also with the values collected in literature. The specific heat value at solid phase only is reported from [8], despite the available value for the liquid phase also, and where neither the material and methods are described nor if the presented results are collected from another work. At the end, the full available LiOH thermal characterisation performed in 1976 is in [9]. Eventually, to the best of our knowledge, there is no information about the thermal and energy stabilities through thermal cycling experiments. Yet, this knowledge is an absolute requirement for a use of LiOH as a S-PCM in the thermal battery technology. The reason is due to the major well-known drawbacks related to the LiOH’s decomposition into Li_2_O as well its reactivity with CO_2_. As reported in [10] and detailed in [6], the LiOH decomposition process steps are closely related to the water presence that behaves both like a reactant and a product. In addition to its hygroscopic behaviour, LiOH corrosive nature and the creeping phenomenon observed when in liquid phase have prevented its use until now as a TES material. However, the work carried on Li_4_Br(OH)_3_ peritectic compound, *-i.e.* on the interaction between LiOH and LiBr to understand its synthesis’ mechanism, shed light on some LiOH’s thermal dynamic behaviour that helped us then to characterise it.

# References:

[1] Y. U. Baïkov, *“Self-diffusion of Lithium, Hydrogen, and Oxygen ions in crystalline Lithium Hydroxide”*, Physics of the Solid State, 52, pp.: 2044-2057, 2010.

[2] L.V. Gurvich, G. A. Bergman, L. N. Gorokhov, V. S. Iorish, V. Ya Leonidov, V.S. Yungman, *“Thermodynamic properties of alkali metal hydroxides. Part 1. Lithium and Sodium hydroxides”*, Journal of Chemistry Reference Data, 25, 1996.

[3] T. Hong-Wei, *“Drawing and application of phase diagram related to eutectic mixed lithium salt ”*, Acta Physico-Chimica Sinica, 23, pp.: 1265-1268, 2007.

[4] J. M. Kiat, G. Boemare, B. Rieu, D. Aymes, *“Structural evolution of LiOH: evidence of a solid-solid transformation toward Li_2_O close to the melting temperature”*, Solid State Communications, 108, pp.: 241-245, 1998.

[5] Y. Takahashi, M. Kamimoto, Y. Abe, R. Sakamoto, K. Kanari, T. Ozawa*, “Investigation of latent heat-thermal energy storage materials. IV. Thermoanalytical evaluation of binary eutectic mixtures of NaOH with LiOH or KOH”*, Thermochimica Acta, 121, pp.: 193-202.

[6] P. Legros, E. Lebraud, F. Achchaq, “*Li4BR(OH)3 microstructure monitoring over its synthesis to tackle the lithium-based salts exploitation challenges as advanced phase change materials for storage technologies”,* Materials and Design, 196, 109160, 2020.

[7] A. G. Fernandez, L. Gonzalez-Fernandez, Y. Grosu, J. Labidi, “*Physicochemical characterization of Phase Change Materials for industrial waste heat recovery applications*”, Energies, 15, 3640, 2022.

[8] A. Raznoshinskaia, I. Troyanoskaya, V. Kozminykh, “*Heat-storing phase change materials: influence of thermophysical properties on stabilization of exhaust temperature*”, MaterialsToday: PROCEEDINGS, 19, pp. 1831-1834, 2019.

[9] R. P. Tye, J. G. Bourne, A. O. Desjarlais, “*Thermal energy storage material-Thermophysical property measurement and heat transfer impact*”, Dynatech Report n° 1503, NASA Report n° NASA-CR-135098, 1976.

[10] U. Wietelmann, M. Steinbild, “*Lithium and lithium compounds*”, Encyclopedia of Industrial Chemistry, Wiley-VCH Verlag GmbH & Co., pp.: 1-36, 2013.
